# The *PPARGC1A *Gly482Ser polymorphism is associated with left ventricular diastolic dysfunction in men

**DOI:** 10.1186/1471-2261-8-37

**Published:** 2008-12-11

**Authors:** Erik Ingelsson, Louise Bennet, Martin Ridderstråle, Marianne Söderström, Lennart Råstam, Ulf Lindblad

**Affiliations:** 1 Department of Medical Epidemiology and Biostatistics, Karolinska Institutet, Stockholm, Sweden; 2 Department of Clinical Sciences, Community Medicine, Lund University, Malmö University Hospital, Malmö, Sweden; 3 Department of Clinical Sciences, Clinical Obesity, Lund University, Malmö University Hospital, Malmö, Sweden; 4 Department of Cardiology, Skaraborg Hospital, Skövde, Sweden; 5 Skaraborg Institute, Skövde, Sweden; 6 Department of Public Health and Community Medicine/Primary Health Care, The Sahlgrenska Academy at Gothenburg University, Sweden

## Abstract

**Background:**

The Gly482Ser polymorphism in peroxisome proliferator-activated receptor gamma coactivator-1 alpha (*PPARGC1A*) has been demonstrated to be associated with diabetes, obesity and hypertension, all of which are important risk factors for left ventricular diastolic dysfunction.

**Methods:**

The *PPARGC1A *Gly482Ser polymorphism was genotyped in a community-based cohort of 499 men and 533 women, who also underwent an echocardiographic examination to determine their left ventricular diastolic function. The association between the polymorphism and the presence of diastolic dysfunction was evaluated using logistic regression models.

**Results:**

The Ser allele of the *PPARGC1A *Gly482Ser polymorphism was significantly associated with a lower risk of diastolic dysfunction in men, but not in women. In a model adjusting for potential confounders (age, body mass index, leisure time physical activity, hypertension and diabetes) the results were still significant and substantial (odds ratio 0.13, 95% confidence interval 0.03–0.54, p for trend = 0.004). The results were consistent in a series of models, and they imply a multiplicative, protective effect of the Ser allele, with lower risk of diastolic dysfunction for each copy of the allele.

**Conclusion:**

The Ser allele of the *PPARGC1A *Gly482Ser polymorphism was associated with decreased risk of diastolic left ventricular dysfunction in men, but not in women, in our large community-based sample. It was associated with a substantially decreased risk, even after adjustment for potential confounders. The clinical importance of the findings has to be established in further studies.

## Background

Heart failure is one of the most common, costly, disabling and deadly diseases, with a lifetime risk of about 20% for a person aged 40 years [1]. Left ventricular systolic function is normal in about half of all heart failure patients [2-4], a condition often referred to as diastolic heart failure. No universally accepted measurement defining left ventricular diastolic dysfunction exists but Doppler-derived diastolic filling indexes have been used extensively [5-7]. Hypertension, diabetes and obesity are more important risk factors in patients with diastolic heart failure, as compared to heart failure patients with reduced left ventricular systolic function [3,4,8,9].

Peroxisome proliferator-activated receptor gamma coactivator-1 alpha (PPARGC1A, often referred to as *PGC1α*) is a major regulator of adipocyte, skeletal muscle and liver metabolism [10]. A frequent substitution of a glycine to serine amino acid at residue 482 in human *PPARGC1A *was described in 2001 [11], and has been demonstrated to be associated with diabetes [11-14], hypertension [15-17], and obesity [18,19]. Further, there are animal data to support a direct role for *PPARGC1A *in the development of heart failure [20-22].

We hypothesized that the *PPARGC1A *Gly482Ser (rs8192673) polymorphism would be associated with diastolic dysfunction measured by Doppler-derived diastolic filling indexes. Further, we aimed to examine whether this association was independent of potential confounders, such as known risk factors for diastolic dysfunction.

## Methods

### Subjects

Within the framework of the Skaraborg Project this study includes data from a surveillance of the population in Vara, a small community in a rural area in South-western of Sweden [18,23]. Residents in Vara were randomly selected from the population census register stratified by sex and 5-years age-groups between 30 and 74 years. Individuals below age 50 were over-sampled three times as compared to those who were older. Participation was based on conducting a study visit to the nurse for a physical examination, returning blood samples, and filling in the questionnaires. Between 2001 and 2003, 1811 unrelated participants were accordingly included (participation rate 82%).

These participants were then consecutively invited to a separate visit for an echocardiografic examination of the heart (UCG) and in all 1058 subjects, 515 men and 543 women were successfully examined. The presence or absence of diastolic dysfunction could be determined for 1044 participants (91% of all who were invited to the UCG investigation). The genotyping of the *PGC1a *polymorphism failed in six subjects, another six had anamneses of heart failure while 1032 subjects (499 men and 533 women) remained for the present study.

The research ethical committee in Gothenburg approved the study. A written informed consent was collected both at the baseline examination and before they underwent the UCG.

### Assessment of clinical variables

Known doctors' diagnoses of diabetes, hypertension, hyperlipidemia, angina pectoris, atrial fibrillation and information on past events of hospitalization were collected by the nurses according to standardised forms. Information concerning demography, socio-economy, and lifestyles including physical activity at leisure time and smoking was collected by questionnaires. Leisure time physical activity (LTPA) was divided into four different categories, ranging from sedentary to hard exercise. Those who participated in regular physical activity for at least 2 hours a week were considered to be physically active in leisure time (levels 3 and 4), while the others were considered sedentary (levels 1 and 2). The questionnaires were filled out at the clinic and the nurses gave some assistance when needed.

A standard physical examination by the study nurses included blood pressure readings in a supine position after 5 minutes rest with the arm at heart level to the nearest 2 mm Hg. Blood pressure was always measured twice one minute apart and the mean was used for diagnoses. A diagnosis of hypertension was based on ongoing blood pressure lowering medication for hypertension, or repeated blood pressure levels ≥ 140/90 mm Hg (one or both) [24,25]. Weight (to the closest 0.1 kg) and height (to the closest cm) was measured in light clothes and no shoes. Obesity was characterized by body mass index (BMI) calculated as body weight in kilo divided by the square of the height in meters (kg/m^2^). Obesity was defined as BMI ≥ 30.0 kg/m^2^. Waist and hip circumferences were measured to the closest cm and waist to hip ratio (WHR) was calculated.

Fasting blood samples were drawn in the morning after a 10-hour overnight fast by the lab technician before a standard 75-g oral glucose tolerance test (OGTT) was administered and samples were drawn before the glucose load and after 2 hours [26]. In subjects without a known history of diabetes, a second fasting plasma glucose sample was drawn another day within the next weeks. For this paper, plasma glucose was analysed in fasting and 2 hours after the administration of the 75-g glucose load. Diagnosis of diabetes was confirmed if there was a known history of physician diagnosis, and in new cases when the fasting plasma glucose value was ≥ 7.0 mmol/L (twice) and/or when the 2 hour plasma glucose value in the OGTT was ≥ 11.1 mmol/L [27]. Serum cholesterol was analysed on fasting samples (standard commercial kits). Hypercholesterolemia was defined as serum cholesterol ≥ 6.5 mmol L^-1^.

### Echocardiographic methods

All participants were examined with echocardiography by the same senior cardiologist (MS) using a General Electrics VingMed S 5 System operating with a 3.5 MHz-probe. Measurements for left ventricular calculations were taken from Guidelines of the American Society of Echocardiography [28]. A 2-dimensional echocardiogram was formed from the left parasternal and apical windows for measurements. The ejection fraction was calculated from apical 4- and 2-chamber views with longitudinal systolic shortening mean index/atrioventricular plane displacement technique in addition to semi-quantitative visual estimation method [29,30]. The ventricular diastolic function was based on several parameters (see below) and their respective normal ranges were defined for different age categories [31].

The early and late diastolic velocities and the ratios of these were determined by a pulsed wave Doppler signal at the tips of the mitral valve in the apical 4-chamber view. The E-wave peak (early filling) to A-wave peak (atrial filling), and the ratio was determined. The isovolumetric relaxation time (IVRT), from the closure of the AV-valve to the opening of the mitral valve was also measured. To indirectly measure the distensibility in the left ventricle the deceleration time (DT), the peak E to baseline slope was measured. Tissue Velocity Imaging (TVI) was used in both septum and in the more stable lateral wall to detect pseudo normalization. The patient was also told to do valsalva during the registration.

Diastolic dysfunction was considered when evidence of abnormal left ventricular diastolic relaxation, filling, and diastolic distensibility and diastolic stiffness was found in the presence of normal or mildly reduced left ventricular systolic function (left ventricular systolic ejection fraction (LVEF ≥ 45%) according to recommendations from ESC [32]. For this study, diastolic function was dichotomised as being normal or not.

### DNA extraction and genotyping

As previously published, [18] DNA was extracted from whole blood using QiaGen MiniPrep (QiaGen, Germany) at the DNA/RNA Genotyping Lab, SWEGENE Resource Center for Profiling Polygenic Disease, Lund University, Malmö University Hospital, Malmö, Sweden. The *PPARGC1A *Gly482Ser polymorphism (rs8192673) was genotyped using an allelic discrimination assay (assay on demand) performed with an ABI 7900 system (Applied Biosystems Inc., USA) using the PCR primers; 5'-TGGAGAATTGTTCATTACTGAAATCACTGT-3' (forward) and 5'-GGTCATCCCAGTCAAGCTGTTTT-3' (reverse) and TaqMan MGB probes, Fam-5'-CTATTGACGCAGAAAG-3' and Vic-5'-CTCCTATTGACCCAGAAAG-3'. Genotyping efficiency was 99.8%, and 100% genotyping accuracy was shown when 5% of the samples were re-run. The frequency distribution was in agreement with the Hardy-Weinberg expectations in men and women, jointly and separately (P > 0.97 for all).

### Statistical methods

Statistical analysis was performed using SPSS 14.0 for PC. Standard methods were used for descriptive statistics. Analyses were stratified on sex according to our a priori analysis plan based on the hypothesis that the association between *PPARGC1A *Gly482Ser and diastolic dysfunction could differ by sex. Confounding by age, or by CVD risk factors, was accounted for by stratification or by multivariate analyses. The rural population in Vara is very homogenous [33] and there are thus no population stratification concerns to address. Prevalence's of the complete study population were standardised according to the population in Vara by 5-year age groups. Association between diastolic dysfunction and categorical variables were analyzed by logistic regression and expressed as odds ratios (OR) with 95% confidence intervals (CI). Differences in means between groups were analyzed by general linear models (GLM). All tests were 2-sided and statistical significance was assumed when p < 0.05.

### Statement of responsibility

The authors had full access to the data and take responsibility for its integrity. All authors have read and agree to the manuscript as written.

## Results

Clinical characteristics and *PPARGC1A *Gly482Ser genotype distribution are shown in Table 1. Both women and men were characterized with a high level of overweight and a low level of leisure time physical activity. Diastolic dysfunction was prevalent in 16.5% of the men and 13.0% of the women. The *PPARGC1A *Gly482Ser genotype distributions across different clinical characteristics by sex are demonstrated in Table 2.

**Table 1:** Descriptive characteristics of the study population

Variables (units)	Men N = 499	Women N = 533
Age (years)	50.0(12.2)	50.3(11.9)
Body mass index (kg m^-2^)	27.0(4.4)	27.1(4.4)
Waist circumference (cm)	95.1(11.6)	85.9(11.6)
Waist-hip ratio	0.95(0.07)	0.84(0.07)
Systolic blood pressure (mm Hg)	127(13.9)	123(13.9)
Diastolic blood pressure (mm Hg)	73(9.5)	70(9.5)
Diastolic dysfunction (%)	79(16.5)	58(13.0)
Hypertension (%)	96(19.3)	96(20.6)
Diabetes (%)	38(8.0)	38(8.1)
Obesity (%)	96(19.3)	127(24.8)
Low leisure-time physical activity (%)	307(62.2)	377(72.0)
*PGC1α*Gly482Ser genotypes:		
Gly/Gly (%)	214(42.8)	229(43.2)
Gly/Ser (%)	237(47.6)	243(45.3)
Ser/Ser (%)	48(9.6)	61(11.5)

Data are means (SD) or numbers (%). Means were adjusted for age difference using general linear models (GLM) and proportions were age standardized according to the population in Vara.

**Table 2:** Characteristics of the *PGC1α *Gly482Ser polymorphism, in males (♂) and females (♀)

		Gly/Gly	Gly/Ser	Ser/Ser
(N:♂/N:♀)		(214/229)	(237/243)	(48/61)
Age (years)	♂	51.3 (12.2)	50.7(12.2)	50.8 (12.2)
	♀	51.8 (11.8)	49.4 (11.8)	47.9 (11.8)
Body mass index (kg m^-2^)	♂	26.9 (3.4)	27.2 (3.4)	27.2 (3.5)
	♀	26.8 (5.1)	27.4 (5.1)	27.0 (5.1)
Low leisure-time	♂	125 (59.1)	150 (63.1)	32 (63.3)
physical activity (%)	♀	163 (71.0)	173 (72.8)	41 (72.9)
Systolic blood pressure	♂	128 (14.2)	127 (14.2)	125 (14.2)
(mmHg)	♀	123 (13.4)	121 (13.4)	124 (13.4)
Diastolic blood pressure	♂	74 (9.6)	73 (9.6)	73 (9.6)
(mmHg)	♀	70 (9.4)	69 (9.4)	70 (9.3)
Diastolic dysfunction (%)	♂	42(19.9)	34 (15.5)	3 (5.1)
	♀	29 (11.7)	27 (15.7)	2 (6.0)
Hypertension (%)	♂	44 (21.1)	43 (18.8)	9 (13.7)
	♀	51 (22.2)	33 (17.7)	12 (25.2)
Diabetes (%)	♂	15 (7.4)	19 (9.1)	4 (4.7)
	♀	18 (7.9)	14 (7.2)	6 (9.0)

Data are means (standard deviations).

When stratifying the study population by sex, the Ser allele of the *PPARGC1A *Gly482Ser polymorphism was significantly associated with a lower risk of diastolic dysfunction in men, but not in women (Table 3). The effect was substantial (odds ratio [OR] 0.13–0.19 for Ser/Ser vs. Gly/Gly), statistically significant and consistent in different models including potential confounders (age, body mass index, leisure time physical activity, hypertension and diabetes). To investigate the mode of action further, we examined whether carriers of at least one copy of the Ser allele had an increased risk of diastolic dysfunction as compared to non-carriers (Table 4). In these models, carriage of the Ser allele was associated with a decreased risk of diastolic dysfunction (OR, 0.52; 95% confidence interval 0.29–0.92, p = 0.026 in the fully multivariable-adjusted model). In women, again there was no significant effect of the *PPARGC1A *Gly482Ser polymorphism. Although the results were different for men and women, the interaction between *PPARGC1A *Gly482Ser and sex was non-significant upon formal testing (*p *= 0.19). There was no significant interaction between *PPARGC1A *Gly482Ser and age (*p *= 0.61) or between *PPARGC1A *Gly482Ser and menopausal status (using age 55 in women as a proxy; *p *= 0.24). With a significance level of 0.05, we had 87% (men) and 90% (women) power to detect a quantitative trait locus that accounted for 1.5% of the residual variance.

**Table 3:** Associations between the *PGC1α *Gly482Ser polymorphism and diastolic dysfunction, in males and females.

	Gly/Gly	Gly/Ser	Ser/Ser			*P *for trend
*Males*	N = 214	N = 237	N = 48			
Covariates	OR	OR	(95% CI)	OR	(95%CI)	
Age	1.0	0.70	(0.40 – 1.22)	0.19	(0.05 – 0.72)	0.012
Age, body mass index	1.0	0.64	(0.36 – 1.14)	0.15	(0.04 – 0.61)	0.005
Age, low leisure-time physical activity	1.0	0.72	(0.41 – 1.26)	0.19	(0.05 – 0.72)	0.015
Age, body mass index, low leisure-time physical activity	1.0	0.66	(0.37 – 1.17)	0.16	(0.04 – 0.66)	0.007
Age, hypertension	1.0	0.70	(0.40 – 1.22)	0.19	(0.05 – 0.72)	0.012
Age, diabetes	1.0	0.67	(0.38 – 1.19)	0.16	(0.04 – 0.63)	0.007
Age, hypertension, diabetes	1.0	0.68	(0.38 – 1.20)	0.14	(0.04 – 0.58)	0.007
Age, body mass index, low leisure-time physical activity, hypertension, diabetes	1.0	0.62	(0.34 – 1.13)	0.13	(0.03 – 0.54)	0.004
						
	Gly/Gly	Gly/Ser	Ser/Ser			*P *for trend
*Females*	N = 229	N = 243	N = 61			
						
Covariates	OR	OR	(95% CI)	OR	(95%CI)	
Age	1.0	1.31	(0.68 – 2.55)	0.30	(0.06 – 1.45)	0.533
Age, BMI	1.0	1.22	(0.61 – 2.41)	0.33	(0.07 – 1.60)	0.495
Age, low leisure-time physical activity	1.0	1.39	(0.71 – 2.73)	0.31	(0.06 – 1.49)	0.603
Age, body mass index, low leisure-time physical activity	1.0	1.29	(0.64 – 2.58)	0.33	(0.07 – 1.62)	0.550
Age, hypertension	1.0	1.31	(0.67 – 2.53)	0.30	(0.06 – 1.47)	0.529
Age, diabetes	1.0	1.38	(0.70 – 2.72)	0.33	(0.07 – 1.60)	0.622
Age, hypertension, diabetes	1.0	1.68	(0.82 – 3.44)	0.32	(0.06 – 1.59)	0.777
Age, body mass index, low leisure-time physical activity, hypertension, diabetes	1.0	1.60	(0.77 – 3.35)	0.35	(0.07 – 1.69)	0.740

Data presented as odds ratios (OR) with 95% confidence interval (CI).

**Table 4:** Associations between Ser carriage and the *PGC1α *Gly482Ser polymorphism and diastolic dysfunction, in males and females.

	Gly/Gly	Gly/Ser + Ser/Ser		*P*
*Males*	N = 214	N = 285		
Diastolic dysfunction, N (%)	42 (19.9)	37 (13.8)		
Covariates	OR	OR	(95% CI)	
Age	1.0	0.59	(0.34 – 1.02)	0.059
Age, body mass index	1.0	0.54	(0.31 – 0.94)	0.031
Age, low leisure-time physical activity	1.0	0.60	(0.35 – 1.05)	0.073
Age, body mass index, low leisure-time physical activity	1.0	0.55	(0.32 – 0.97)	0.038
Age, hypertension	1.0	0.58	(0.33 – 1.01)	0.054
Age, diabetes	1.0	0.56	(0.32 – 0.98)	0.043
Age, hypertension, diabetes	1.0	0.56	(0.32 – 0.99)	0.044
Age, body mass index, low leisure-time physical activity, hypertension, diabetes	1.0	0.52	(0.29 – 0.92)	0.026
			
	Gly/Gly	Gly/Ser+Ser/Ser		*P*
*Females*	N = 229	N = 304		
Diastolic dysfunction, N (%)	29 (11.7)	29 (13.6)		

Covariates	OR	OR	(95% CI)	
Age	1.0	1.08	(0.57 – 2.04)	0.825
Age, body mass index	1.0	1.01	(0.52 – 1.96)	0.968
Age, low leisure-time physical activity	1.0	1.13	(0.59 – 2.16)	0.717
Age, body mass index, low leisure-time physical activity	1.0	1.06	(0.54 – 2.07)	0.867
Age, hypertension	1.0	1.27	(0.64 – 2.50)	0.492
Age, diabetes	1.0	1.13	(0.59 – 2.16)	0.772
Age, hypertension, diabetes	1.0	1.29	(0.65 – 2.56)	0.463
Age, body mass index, low leisure-time physical activity, hypertension, diabetes	1.0	1.24	(0.62 – 2.51)	0.539

Data presented as odds ratios (OR) with 95% confidence interval (CI).

## Discussion

### Principal Findings

In this community-based sample from the general population, the *PPARGC1A *Gly482Ser polymorphism was associated with the presence of diastolic left ventricular dysfunction in men, but not in women. More specifically, the Ser allele was associated with a substantially decreased risk of diastolic dysfunction, also after adjustment for potential confounders, such as age, body mass index, leisure time physical activity, hypertension and diabetes. The results were consistent in a series of models, and they imply a multiplicative, protective effect of the Ser allele, with lower risk of diastolic dysfunction for each copy of the allele. In the present study, the prevalence of diastolic dysfunction was somewhat higher in men than in women. Left ventricular systolic function is more commonly preserved in women with heart failure [2,3], but the prevalence of diastolic dysfunction in the general population has been established to be higher in men than women [9].

### Comparisons with Previous Studies

Recently, other *PPARGC1A *polymorphisms were shown to be associated with presence of self-reported cardiovascular disease, and it was suggested that this association was mediated via DNA damage [34]. Also, the *PPARGC1A *Gly482Ser polymorphism has been associated with hypertrophic cardiomyopathy [35]. Further, previous studies have demonstrated the Ser allele of the *PPARGC1A *Gly482Ser to be associated with an increased risk of diabetes [11-13], but a recent meta-analysis have implied only a modest role for the polymorphism in the development of diabetes [14]. Moreover, the Ser allele has been associated with obesity in some studies [18,19], whereas other have failed to find significant associations between the *PPARGC1A *Gly482Ser polymorphism and various body fat measures and the metabolic syndrome in different study populations [36-38]. The *PPARGC1A *Gly482Ser polymorphism has also been associated with hypertension in previous studies [15-17]. In two of these studies, the Ser allele conferred a lower risk of hypertension [15,16], whereas one study demonstrated an increased risk of hypertension for carriers of the Ser allele [17]. However, the latter study consisted only of subjects with diabetes; hence this inconsistency could be a result of a gene-environment interaction. A recent large meta-analysis comprising of 13 949 individuals from 17 studies, of which 6 042 were from previously unpublished datasets, failed to find an association of the *PPARGC1A *Gly482Ser polymorphism with hypertension overall. However, they reported higher systolic and diastolic blood pressure in younger individuals (< 50 years of age) with the Ser allele, whereas there were no such associations in elderly [39].

To the best of our knowledge, this is the first epidemiological study of associations between the *PPARGC1A *Gly482Ser polymorphism and diastolic dysfunction. We observed a protective effect of the Ser allele, with lower risk of diastolic dysfunction for each copy of the allele in men. This contrasts with prior findings that the Ser allele is associated with higher risk of diabetes, [11-13], but agrees with findings that it is associated with lower risk of hypertension, [15,16] both of which are major risk factors for diastolic dysfunction. In our investigation, the findings were independent of the presence of hypertension and diabetes. Further studies are needed to confirm our results and to further elucidate the role of *PPARGC1A *in the development of diastolic dysfunction.

### Possible Mechanisms

PPARGC1A is a key metabolic protein, which induces and coordinates gene expression of metabolic genes through specific interaction with transcription factors that bind to the promoter regions the genes. It stimulates mitochondrial biogenesis and is a major regulator of adipocyte, skeletal muscle and liver glucogenesis metabolism [10].

It is plausible that PPARGC1A levels and *PPARGC1A *gene polymorphisms could be associated with cardiac function, as *PPARGC1A *is an important regulator of metabolic pathways, and as heart failure and diastolic dysfunction can be caused by metabolic disturbances, such as diabetes and insulin resistance [40-42], obesity [8,9] and hypertension [1]. Thus, the association between *PPARGC1A *and diastolic dysfunction might be secondary, through these intermediate phenotypes. However, as the association between the polymorphism and diastolic dysfunction was evident also after adjustment for these potential confounders, our results imply a more direct effect of *PPARGC1A *on the cardiac function. The Ser allele of the *PPARGC1A *Gly482Ser polymorphism has been demonstrated to be associated to lower *PPARGC1A *mRNA expression [43]. This could be a potential explanation of our findings of a protective effect of the Ser allele on diastolic dysfunction, as animal studies have indicated high PPARGC1A levels to be involved in the development of cardiac dysfunction. Forced expression of PPARGC1A has been demonstrated to activate mitochondrial biogenesis and metabolism in cardiac myocytes in vitro, and cardiac-specific over-expression of PPARGC1A in transgenic mice has led to mitochondrial proliferation and loss of sarcomeric structure in cardiac myocytes, leading to a dilated cardiomyopathy [20].

In the present study, we found associations between the *PPARGC1A *Gly482Ser polymorphism and diastolic left ventricular dysfunction in men, but not in women. The interaction between *PPARGC1A *Gly482Ser and sex was non-significant upon formal testing, which could be due to low statistical power to detect interactions. However, the analyses were stratified on sex according to our a priori analysis plan based on the hypothesis that the association between *PPARGC1A *Gly482Ser and diastolic dysfunction could differ by sex. Previous studies on this polymorphism and different metabolic traits have also reported gender differences [15-19]. There are several possible reasons for this. A recent study demonstrated that the PPARGC1A mRNA levels were lower in women than in men [43]. Moreover, PPARGC1A interacts with estrogen receptors and enhances their transcriptional activity [44]. Estrogen receptors are expressed in vascular endothelial and smooth muscle cells, as well as in myocardial cells, and there are gender differences in expression [45]. Estrogen and estrogen receptors have repeatedly been demonstrated to be involved in cardiovascular disease and several recent animal studies have implied a role for estrogen receptors in the development of cardiac dysfunction [46-48]. Also, a recent study of patients with hypertrophic cardiomyopathy demonstrated polymorphisms in the estrogen receptor alpha (*ESR1*) gene, as well as in the androgen receptor (*AR*) gene to be associated with left ventricular wall thickness in men, but not in women [49]. Similarly, another recent study reported a 1.8-fold increase in estrogen receptor alpha mRNA levels in patients with end-stage dilated cardiomyopathy [50].

### Strengths and limitations

The strengths of this study include the large community-based population, the high participation rate and well-characterized cohort. There are also some limitations of our study. The study has an unknown generalizability to other ethnic groups. On the other hand, we circumvent the powerful effects of population stratification. Moreover, as we only genotyped one polymorphism in the *PPARGC1A *gene, there might be other polymorphisms with an even stronger association to diastolic dysfunction. Finally, we acknowledge the risk of false positive findings and the lack of an independent replication sample for our findings.

## Conclusion

In conclusion, the *PPARGC1A *Gly482Ser polymorphism was associated with the presence of diastolic left ventricular dysfunction in men, but not in women, in our large community-based sample. The Ser allele was associated with a substantially decreased risk of diastolic dysfunction, also after adjustment for potential confounders as age, body mass index, leisure time physical activity, hypertension and diabetes. Since the Ser allele has previously been associated with decreased expression of *PPARGC1A*, it is possible that this is one explanation for the protective effect observed here. Meanwhile, decreased expression of *PPARGC1A *in skeletal muscle has been associated with an increased risk for type II diabetes. The complexity of the role of *PPARGC1A *in cardiovascular disease certainly warrants further research and the clinical importance of the present findings has to be established in further studies. These potentially hypothesis-generating findings could stimulate further exploration in other studies.

## Competing interests

The authors declare that they have no competing interests.

## Authors' contributions

EI, conceived and designed the current study, participated in the analyses and the interpretation of the data, and wrote the paper.

LB, performed the statistical analyses, participated in interpreting the data, and participated in writing the paper.

MR, was in charge of the molecular genetic analyses, participated in preparing and interpreting the data, and in writing the paper.

 MS, made and evaluated all echocardiograms, participated in preparing and interpreting the data, and in writing the paper.

LR, initated the Skaraborg project, participated in interpreting the data, and in writing the paper.

UL, initiated the Skaraborg project, conceived and designed the current study, participated in interpreting the data, and participated in writing the paper.

## Pre-publication history

The pre-publication history for this paper can be accessed here:


